# Effects of gamma radiation on fetal development in mice

**Published:** 2016-04

**Authors:** Tahere Dehghan, Hossein Mozdarani, Arezoo Khoradmehr, Seyed Mehdi Kalantar

**Affiliations:** 1 *Shahid Beheshti University, Tehran, Iran.*; 2 *Department of Medical Genetics, Faculty of Medical Sciences, Tarbiat Moddares University, Tehran, Iran.*; 3 *Research and Clinical Center for Infertility, Shahid Sadoughi University of Medical Sciences, Yazd, Iran.*

**Keywords:** *Gamma rays*, *Whole body irradiation*, *PMSG-HCG*

## Abstract

**Background:**

Many cancer patients receive radiotherapy which may lead to serious damages to the ovary storage and the matrix muscle state. Some of these patients may admit to infertility clinics for having pregnancy and on the other hand hormonal administration for superovulation induction is a routine procedure in assisted reproduction technology (ART) clinics.

**Objective:**

This study aimed to investigate fertility and fetuses of hormone treated super ovulated female mice who had received whole-body gamma irradiation before mating.

**Materials and Methods:**

Female mice were randomly categorized into a control group and 3 experimental groups including: Group I (Irradiation), Group II (Superovulation), and Group III (Superovulation and Irradiation). In hormone treated groups, mice were injected with different doses of pregnant mare's serum gonadotropin (PMSG) followed with human chorionic gonadotropin (HCG). Irradiation was done using a Co-60 gamma ray generator with doses of 2 and 4 Gy. Number of fetuses counted and the fetus’s weight, head circumference, birth height, the number of live healthy fetuses, the number of fetuses with detected anomalies in the body, the sum of resorption and arrested fetuses were all recorded as outcome of treatments.

**Results:**

In the group I and group II, increased radiation and hormone dose led to a decrease in the number of survived fetuses (45 in 2 Gy vs. 29 in 4 Gy for irradiated group) as well as from 76 in 10 units into 48 in 15 units. In the group III, a higher dose of hormone in the presence of a 2 Gy irradiation boosted the slink rate; i.e. the number of aborted fetuses reached 21 cases while applying the dose of 15 Iu, whereas 6 cases of abortion were reported applying the hormone with a lower dose. Among different parameters studied, there was a significant difference in parameters of weight and height in the mouse fetuses (p=0.01).

**Conclusion:**

The data indicated that use of ovarian stimulating hormones in mice that received pre mating gamma irradiation did not significantly increase the pregnancy rates.

## Introduction

A great deal of progress has been made in regard with the cancer treatment, life expectancy and longevity of those patients with malignancy. On the other hand, regarding the damage that may be caused by cancer treatment (chemotherapy or radiotherapy) on female patients' fertility, preserving fertility capability of such patients is of great interest. Therefore, an effective solution necessitates to be put forward to preserve the mentioned patients’ fertility power, and the patients should be informed before being treated via chemotherapy or radiotherapy (1). Such cancer treatment procedures as chemotherapy and radiotherapy can exert serious damaging influence on ovarian reserve which leads to follicular pool reducing (2).

The effects of radiotherapy on ovarian reserve relies on factors including the patient's age, the dose received by ovaries, and simultaneous use of chemotherapy. In addition to ovarian failure, the uterus might be damaged by radiotherapy, as well. This adverse impact is notably made by altering its muscular vascularization as well as decreasing uterine volume, both of which lead to of fetus growth during the pregnancy in regard with those females who underwent radiotherapy during their childhood (3). The atrophy of endometrial glands and stroma are likely to be observed in radiotherapy of uterus and cervix receiving high doses of radiation. Moreover, normally ulceration and necrosis remain for several months, which may be replaced by dense collagen deposition. The cervix gets quite atrophic and loses its elasticity specifically in older patients. It is worth mentioning besides uterine and ovary damage, irradiation may increase disorders probability of placenta attachment such as placental accrete or placental percreta (4).

The studies conducted on female survivors of Wilms tumor, who had underwent radiotherapy during their childhood demonstrated that they are at increased risk of preterm labor, fetal malposition, and having premature infants of low birth weight. Decreased uterine elasticity and its volume induce myometrial changes through the irradiation, such as fibrosis, which may lead to preterm labor or premature delivery (5). Hence, these issues should be taken into consideration in patients’ obstetrical management of their pregnancies.

Norwitz *et al* showed reduced uterine volume and impaired uterine blood flow in patients who underwent total body irradiation for bone marrow transplantation (radiation exposure received by patient was much less than dose received by patients undergoing abdominal or pelvic radiotherapy). Although same radiation effects were observed on uterine, the severity of injuries was lower due to low doses of irradiation (6). Therefore, these alternations in uterus can lead to increased risk of uterine rupture (7).

A study carried out on females with an irradiation history beyond 45 Gy in pelvis region in adulthood has revealed a high risk of infertility for such women. Hormone therapy plus concurrent use of pentoxifylline and tocopherol have been recommended as a solution in this study in order to ameliorate the performance of uterus (8). Since young female patients' fertility may be impaired as result of serious side effects of radiotherapy, several remedies have been proposed to preserve patients’ fertility after treatment such as ovarian tissue cryopreservation, egg freezing and embryo freezing (9). 

It should be noted that there are always some patients, who did not apply any of these remedies before undergoing radiotherapy. Hence, this question may arise that "are ovarian stimulation hormones likely to bring pregnancy for these patients?” The current study aimed to investigate the mice fertility who had received Gamma irradiation before pregnancy. As a matter of fact, the pregnancy process, pregnancy period up to 18^th^ day, as well as fetuses was evaluated within female irradiated mice for which ovarian stimulation hormones have been utilized before pregnancy. 

More prominently, regarding effect of irradiation on uterus, influence of ovarian stimulating hormones on uterus, and its role in maintaining pregnancy from implantation to delivery, this study intended to investigate the still born of female mice before delivery that is to say 18^th^ day of pregnancy, whereas previous study conducted by author of present study explored the mice with same circumstances on 3^th^ day of pregnancy (before the implantation) (10).

## Materials and methods

This experimental study was conducted in Yazd Research and Clinical Center for Infertility. The study have been approved by the Ethical Committee of Yazd Research and Clinical Center for Infertility (Ref no; 284).

**Animals**

The experimental and control group consisted of 45 six to eight-weeks-old female mice, purchased from Yazd Research and Clinical Center for Infertility, Iran. Female mice were kept in separate cages (5 per cage) for at least 1 wk before conducting the experiment, so that they would get used to laboratory environment. Mice were kept in an environment with 12 hr light/dark and temperature of 20-24^o^C and 60-70% of humidity. Female mice were fed with standard breeding granulated diet and water ad libitum. After a week, female mice were randomly classified into a control group and 3 experimental groups including: Group I (Irradiation), Group II (Superovulation), Group III (Superovulation and Irradiation). The control group were exposed to neither hormone nor irradiation before mating.

**Group I (Irradiation)**

Female mice in irradiation group received whole body irradiation of 2 Gy or 4 Gy gamma-rays. generated from a cobalt-60 source (Theratron II, 780 C, Canada) at a dose rate of 55 cGy/min with sample source distance (SSD)=82 cm. The field size of irradiation was 10×10 cm in a room at ambient temperature (23±2)^o^C. In order to examine the effect of irradiation, the female mice were exposed to total body irradiation of 2 and 4 Gy gamma rays, in morning. In the same evening, each female mouse was coupled with a non-irradiated male mouse in a cage. 

The following morning, female mouse was checked for presence of vaginal plug and those mice with positive plug were considered as pregnant. On 18^th^ day of pregnancy, 10 pregnant female mice; five from 2Gy group and five from 4Gy group were sacrificed through cervical dislocation. Eventually, the fetuses were removed from the uterine horn for further examinations.

**Group II (Superovulation)**

In superovulation group, female mice received an intraperitoneal injection (IP) of 10 or 15 units of Pregnant mare's serum gonadotropin, (PMSG; IBSA. Holland) hormone followed by injection of 10 or 15 Iu of human chorionic gonadotropin (HCG; Organon, Holland) with 42-48 hr intervals. After HCG injection, female mouse was mated with male one. 

Next morning, female mice were checked for presence vaginal plugs (VP) and the VP-positive female mice were considered as pregnant. Pregnant mice were kept safe until 18^th^ day of pregnancy, when 10 pregnant female mice, five from 10 Iu group and five from 15 Iu group were sacrificed through cervical dislocation and eventually, fetuses were collected from uterine horn for further examinations. 15 Iu hormone injection was used in order to study the effect of hormone overdose on mice while irradiation impaired a great number of ovarian follicles. Therefore, within both group II and group III, the identical hormone level of 10 Iu and the overdose level of 15 Iu were applied (12).

**Group III (Superovulation and Irradiation)**

In the superovulation and irradiation group, female mice received total body irradiation alone via a dose of 2 Gy or 4 Gy almost 12-18 hr prior to HCG injection (30-36 hr after PMSG injection) (13). Each female mouse was made to mate with a non-irradiated male mouse. In following morning, female mice were investigated in regard with their positive vaginal plug. Those positive-plug mice were considered as pregnant. 

On the 18^th^ day of pregnancy, the retrieve fetuses of 20 pregnant female mice were investigated. These 20 mice were classified into four groups of 5 female mice consisting of 15 Iu hormone mice which were exposed to 2 Gy irradiation, the 15 Iu hormone mice which were exposed to 4Gy irradiation, 10 Iu hormone mice which were exposed to 2Gy, and 10 Iu hormone mice who were exposed to 4 Gy. Eventually, fetuses were retrieved from uterine horn. 

**Embryos retrieve on the eighteenth day of pregnancy**

Pregnant female mice were sacrificed through cervical dislocation in the 18^th^ morning of pregnancy in all agroups. An incision was made in abdomen and fetuses were removed from amniotic sac. Eventually, fetus’s weight, head circumference, birth height, number of live healthy fetuses, number of fetuses with detected anomalies in body, sum of resorption and arrested fetuses were all recorded; weight was measured with scales. As well as head circumference, the birth height of fetus were measured with caliper. Head circumference was measured with C=π{3(A+B)-[(3A+B) (A+3B)] 1/2} formula, (A, B: Big and small circumference).

**Statistical analysis**

The study data were analyzed using SPSS software version 16.0 (Chicago, USA) and nonparametric statistical tests of Kruskal-Wallis, Mann-Whitney and analysis of variance (ANOVA). p˂0.05 was considered as significant. 

## Results

The total number of live and aborted fetuses (total number of resorption fetuses and arrested fetuses), removed from each groups in 18^th^ day of pregnancy is demonstrated in table I. As table I reports, in general in control group, 59 live and 3 aborted fetuses were collected from 5 mother mice, whereas total number of live fetuses obtained from mother mice was 45 in group exposed to 2 Gy. By irradiation increasing to 4Gy, total number of live fetuses reached to 29. Simultaneously, abortion rate increased from 8-16 cases. In group II, increasing superovulation hormone dose led to decreasing of total number of live fetuses from 76 in the 10 Iu hormone to 48 in the 15 Iu. Furthermore, abortion rate in the 5 mother mice who received 10 units of hormones was 16, while its rate in other group receiving 15 Iu hormone was 10.

In group III, on 18^th^ day of pregnancy, regarding 5 female mice with 2Gy + 10 Iu of hormone, total number of live fetuses reached to 49 and abortion rate involved 6. Besides, for the group, which received the same irradiation but 15 Iu of hormone, total number of live fetuses was reported as 48 and abortion rate was 21. On the other hand, regarding 5 female mice with 4Gy + 10 Iu of hormone, the total number of live fetuses reached to 48 and the abortion rate was 10, while for 5 female mice which received same rate of irradiation but 15 Iu of hormone, the total number of live fetuses reached to 58 and the abortion rate was 17. 

The parameters pertaining to live fetuses removed from amnion sac were evaluated as well, including weight, head circumference, and birth height of embryos (Table I). Statistical analysis demonstrated that among all the parameters including the total number of live fetuses in 18^th^ day of pregnancy, the abortion rate, fetus’s weight, head circumference, and birth height only the two parameters of fetus’s weight and birth height were significantly different among groups (p=0.01) . Furthermore, comparison between groups revealed that, weight of fetuses in group exposed to 4Gy irradiation increased significantly compared to weight of those fetuses in 2 Gy + 15 Iu, 4 Gy + 10 Iu and 4 Gy + 15 Iu groups.

In addition, birth height of fetuses in 4Gy-irradiation group demonstrated a significant difference with birth height of fetuses in a 4 Gy +10 Iu group. In addition, fetuses had been examined according to atlas of anatomy, in terms of macroscopic changes and also the existence of anomaly in face, hands and other parts of body (14). However, in a 4Gy group, only two fetuses had a short neck and protruding in back of their skull. On the other hand, there were also two cases in 4Gy +15 Iu group which were reported to have same anomalies. 

**Figure 1:**
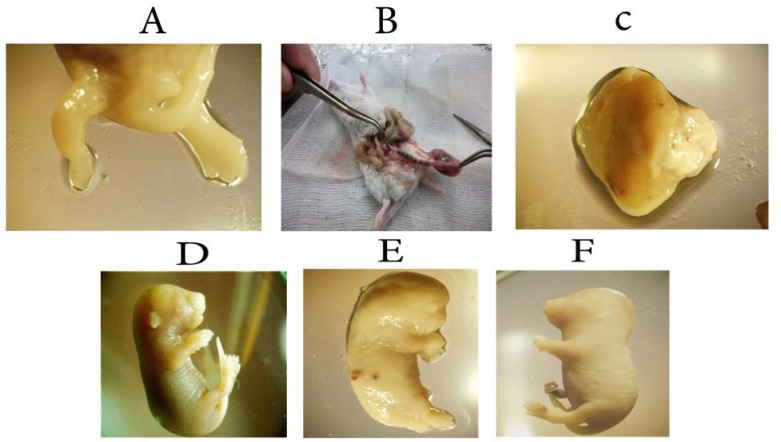
A: Dead fetus with foot anomaly (the absence of toes) (2+15 group), B: Resorbtion fetus (4-Gy group), C: Arrested fetus. (2 Gy group), D: Live embryo (control group), E: Immature embryo (10 Iu group), F: Embryo with short neck and protruding in the back of her skull (4-Gy group) (20×).

Besides, a dead fetus with foot anomaly (absence of toes) in 2 Gy +15 Iu group, two immature fetuses in 10 Iu hormone group, an immature fetus in control group and another in a 4 Gy +15 Iu group. Figure 1 illustrate a dead fetus with foot anomaly, resorbtion fetus, arrested fetus, live fetus, immature fetus, and a fetus with short neck and protruding in the back of her skull.

**Table I:** The total number of live (p=0.52), aborted fetuses (p=0.54) (total number of resorption and arrested fetuses), the fetuses' weight, head circumference, birth height after removal of fetuses from the uterine horn of mousses in the eighteenth day of pregnancy.

	**Control**	**2Gy**	**4Gy**	**10Iu**	**15Iu**	**2-10Iu**	**2-15Iu**	**4-10Iu**	**4-15Iu**
The total number of live fetuses	59	45	29	76	48	49	48	44	58
Aborted fetuses	3	8	16	16	10	6	21	10	17
X(gr)±SD embryo’s weight*	0.89±0.24	0.92±0.15	1.17±0.4	0. 89±0.1	0.85±0.14	0.93±0.12	0. 7±0.14	0. 65±0.2	0.71±0.19
X(mm)±SD head circumference**	0.91±0.07	0.81±0.11	0.94±0.12	0. 9±0.03	0. 9±0.11	0. 9±0.07	0. 79±0.1	0.73±0.21	0.79±0.16
X(mm)±SD birth height *	1.9±0.14	1.98±0.13	2.21±0.35	1.87±0.18	1.84±0.18	1.94±0.14	1.79±0.21	1.64± .17	1.76±0.24

Using ANOVA and Kruskal-Wallis, Mann-Whitney tests were calculated.

* p=0.01

** p=0.12

## Discussion

The female mice, undergoing pelvic irradiation, are more likely to suffer from side effects pertaining to pregnancy as spontaneous miscarriages, preterm labor, low-birth-weight infants, and placental disorders. These complications might be assigned to such elements as reduced uterine elasticity due to myometrial fibrosis, decreased uterine volume, uterine vasculature injury and endometrial damage. However, severity of such damages relies on following factors: applied radiation dose, irradiation place, and patient age at treatment time (15). Although ovarian stimulation offers a great deal of merits specifically in assisted reproductive technology (ART), it might reveal adverse effects on oogenesis, embryo quality, endometrial receptivity and perinatal outcomes (16).

Some scholars demonstrated that higher rate of post implantation mortality was observed in super ovulated mice. Furthermore, live fetuses were reported to have developmental retardation as well as reduced fetal weight. In a nutshell, it can be inferred that there is probable association between detrimental effects resulted from superovulation and changes in maternal milieu of super ovulated mice. Hence, these changes may adversely lead to uterine receptivity, implantation, and fetal development (17).

Irradiation can decrease the number, birth weight and birth height of live fetuses on 18^th^ day of pregnancy. Moreover, it resulted in an increase in number of aborted fetuses. Birth weight decrement was also observed in hormone group. In group I, a sharp decline in number of live fetuses and an increase in abortion rate was observed. Mentioned disorders were caused by negative effects of irradiation on ovary and uterus. Some studies proposed that irradiation can decrease an ovarian follicular reserve. 

As a result, fetus formation decreases and uterus loses some of its ability to preserve fetus (18). Studies concluded that increasing the irradiation dose can intensify negative effects (19). Hence, in high doses of 4Gy irradiation, the severe depletion of ovary resulted in fewer numbers of live fetuses contrast with 2 Gy irradiation. Thus, the fetuses in 4Gy group revealed higher birth weight and height than those of 2Gy group. In group II, utilization of 10 IU hormone leading to an increase in live fetuses’ on 18^th^ day of pregnancy compared to control group. A number of scientists have stated that first phase of hormone injection plays the same role as FSH hormone secretion in body. 

It can stimulate follicles’ growth and thus, in contrast to normal situation, more follicles become mature. In second phase of hormone injection, HCG releases the oocytes that play same role as body LH hormone . (20). However, within female mice, the number of live fetuses which received 15 IU of hormone was less than that of female mice, which received 10 IU of hormone Nonetheless, abortion rate demonstrated no significant difference in two groups (Table I). Some studies have revealed that superovulation hormones decrease the rate of fetus implantation, whereas it can increase the abortion rate in this phase (21).

Circulating steroids can be aggrandized as the result of external administration of gonadotrophins (FSH and LH hormones) which, in turn, may reduce implantation rate. This decrease can be caused by lack of synchrony between fetuses and endometrium at implantation time. Furthermore, oocyte and fetus quality, oviductal and uterine environment might be affected. Mean of live fetuses’ weight and height in 15 Iu group was less compared to 10 Iu group. Yet, comparing this parameter between pairs among groups did not report this decrease as significant (Table I). 

Some studies have shown that mean of weight and height were reported less than those of control group mice in regard with fetuses transplanted into stimulated mice. This decrease can be due to impaired implantation and gestation (22). The reduction of birth weight and height was due to negative effects of superovulation hormone on fetus growth due to ART (23).

In the superovulation + 2Gy irradiation group (III), there was no significant difference between numbers of live fetuses in mice receiving 10 Iu of hormone with ones who received 15 Iu. Indeed, utilization of higher doses led to more abortions and did not cause any increase innumber of live fetuses. Some studies have concluded that overdose can lead to pre-implantation abortion (24). The mean of live fetuses’ weight and height in 15 Iu group was less than that of 2Gy + 10 Iu group (Table I). However, given the negative effects of hormones on fetal development in uterine, the fetuses revealed less maturation compared to fetuses of 2Gy + 10 Iu group. It is worth mention that irradiation exerted a decreasing influence on fetus weight. Both negative factors resulted in birth weight decrease (25).

In superovulation + 4Gy irradiation group, no significant difference was observed in regard with numbers of live fetuses and abortion between group 15 Iu + 4Gy and group 10 + 4 (Table I). Depletion of ovarian follicular reserve with 4Gy irradiation resulted in reduced effect of superovulation hormone on maturation of follicles as well as on number of released eggs. The mean of live fetuses’ weight and height in 4Gy + 15 Iu group was slightly better than 4Gy + 10 Iu group (Table I). The severe deletion in vary caused by 4Gy dose resulted in maintenance of mature oocytes. Through the irradiation, mature oocytes showed more resistance than immature follicles, therefore, irradiation may cause fewer damages to them. Mature oocytes have capacity to live and will have better chance of surviving and growing (26). Some studies have demonstrated that genetic damage of oocyte caused by radiotherapy results in serious delivery complications and fetus anomalies within patients who underwent radiation therapy before pregnancy (27-29). 

In the current study, the 18-day-old embryos were examined macroscopically; whereas, congenital diseases diagnosis requires microscopic investigations, which were not, taken into consideration as an objective in present study. The relative small number of malformations occurred in this study cannot be considered as sufficient evidence to attribute these negative effects to irradiation, hormone or both. Anyway, more comprehensive studies need to be conducted.

Overall, it can be concluded that using superovulation hormones to increase pregnancy chance after lower doses of radiation such as 2Gy can increase number of live fetuses, it can decrease the quality and health of live fetuses. In truth, this negative influence gets aggravated via applying higher doses of hormone. Even for higher doses such as 4Gy, there exist some post-irradiation negative effects of hormone usage. Only difference lies in lesser effect of supra-therapeutic doses of hormones compared to therapeutic doses (10 units of hormone). This phenomenon results from severe depletion in ovary occurred by gamma rays.

The findings of current study (examining 18-day-old embryos) demonstrated that use of ovarian stimulating hormones in mice that received pre-mating gamma irradiation did not significantly increase the pregnancy rates.

## Note

***This article extracted from*** ***M.Sc. thesis. (Tahereh Dehghan)***

## References

[1] Hyman J H, Tulandi T (2013). Fertility Preservation Options After Gonadotoxic Chemotherapy. Clin Med Insights Reprod Health.

[2] Gracia CR, Sammel MD, Freeman E, Prewitt M, Carlson C, Ray A (2012). Impact of cancer therapies on ovarian reserve. Fertil Steril.

[3] Morice P, Uzan C, Gouy S, Pautier P, Lhomme C, Balleyguier C (2010). Effects of radiotherapy (external and/or internal) and chemotherapy on female fertility. Bull Acad Natl Med.

[4] Cohen LE (2008). Cancer treatment and the ovary: the effects of chemotherapy and radiation. Ann N Y Acad Sci.

[5] Green DM, Lange JM, Peabody EM, Grigorieva NN, Peterson JA, Kalapurakal JA (2010). Pregnancy Outcome After Treatment for Wilms Tumor: A Report From the National Wilms Tumor Long-Term Follow-Up Study. J Clin Oncol.

[6] Norwitz ER, Stern HM, Grier H, Lee-Parritz A (2001). Placenta percreta and uterine rupture associated with prior whole body radiation therapy. Obstet Gynecol.

[7] Winther JF, Boice JDJr, Svendsen AL, Frederiksen K, Stovall M, Olsen JH (2008). Spontaneous abortion in a Danish population-based cohort of childhood cancer survivors. J Clin Oncol.

[8] Teh WT, Stern C, Chander S, Hickey M (2014). The impact of uterine radiation on subsequent fertility and pregnancy outcomes. Biomed Res Int.

[9] Dayal MB (2012). Fertility Preservation Options for Female Cancer Patients: A Systematic Review. J Fert In Vitro.

[10] Dehghan T, Mozdarani H, Khoradmehr A, Kalantar S M, Bakhshandeh M, Bouzarjomehri F (2014). Evaluation of embryo quality after concurrent use of ovarian stimulating hormones and gamma irradiation. Iran J Reprod Med.

[11] Wallace WH, Thomson AB, Kelsey T (2003). The radiosensitivity of the human oocyte. Hum Reprod.

[12] Ertzeid G, Storeng R (2001). The impact of ovarian stimulation on implantation and fetal development in mice. Hum Reprod.

[13] Salimi M, Mozdarani H (2008). Reduction of transgenerational radiation induced genetic damages observed as numerical chromosomal abnormalities in preimplantation embryos by vitamin E. Nukleonika.

[14] Kaufman HM (1992). The atlas of mouse development.

[15] Signorello LB, Mulvihill JJ, Green DM, Munro HM, Stovall M, Weathers RE (2010). Stillbirth and neonatal death in relation to radiation exposure before conception: a retrospective cohort study. Lancet.

[16] Santos MA, Kuijk EW, Macklon NS (2010). The impact of ovarian stimulation for IVF on the developing embryo. Reproduction.

[17] Hashemitabar M, Ghavamizadeh B, Javadnia F, Sadain E (2004). The impact of ovarian stimulation and luteal phase support on embryo quality and implantation process in mice. Iran J Reprod Med.

[18] Morice P, Uzan C, Gouy S, Pautier P, Lhomme C, Balleyguier C (2010). Effects of radiotherapy (external and/or internal) and chemotherapy on female fertility. Bull Acad Natl Med.

[19] Critchley HOD, Wallace WHB (2005). Impact of cancer treatment on uterine function. J Nat Cancer InstMonographs.

[20] Brooke DA, Orsi NM, Ainscough JFX, Holwell SE, Markham AF, Coletta PL (2007). Human menopausal and pregnant mare serum gonadotrophins in murine superovulation regimens for transgenic applications. Theriogenology.

[21] Ertzeid G, Storeng R (2001). The impact of ovarian stimulation on implantation and fetal development in mice. Hum Reprod.

[22] Santos MA, Kuijk EW, Macklon NS (2010). The impact of ovarian stimulation for IVF on the developing embryo. Reproduction.

[23] Ombelet W, Martens G, De Sutter P, Gerris J, Bosmans E, Ruyssinck G (2006). Perinatal outcome of 12,021 singleton and 3108 twin births after non-IVF-assisted reproduction: a cohort study. Hum Reprod.

[24] Wu BJ, Xue HY, Chen LP, Dai YF, Guo JT, Li XH (2013). Effect of PMSG/hCG Superovulation on Mouse Embryonic Development. JIntegr Agr.

[25] Green DM, Sklar CA, Boice JD Jr, Mulvihill JJ, Whitton JA, Stovall M (2009). Failure and Reproductive Outcomes After childhood Cancer Treatment: Results From the Childhood Cancer Survivor Study. J Clin Oncol.

[26] Kashchenko L A (1962). The mechanism of radiation damage in the ovary of the mouse. USSR-Journal.

[27] Stroud JS, Mutch D, Rader J, Powell M, Thaker PH, Grigsby PW (2009). Effects of cancer treatment on ovarian function. Fertil Steril.

[28] Mueller BA, Chow EJ, Kamineni A, Daling JR, Fraser A, Wiggins CL (2009). Pregnancy outcomes in female childhood and adolescent cancer survivors:a linked cancer-birth registry analysis. Arch Pediatr Adolesc Med.

[29] Reulen RC, Zeegers MP, Wallace WH, Frobisher C, Taylor AJ, Lancashire ER (2009). Pregnancy outcomes among adult survivors of childhood cancer in the British Childhood Cancer Survivor Study. CancerEpidemiol Biomark Prev.

